# Epigenetics & modern phenotypes: the biological nonsense of sub-talar neutral

**DOI:** 10.1186/1757-1146-3-S1-O21

**Published:** 2010-12-20

**Authors:** Greg Quinn

**Affiliations:** 1 Holywell Healthcare, Chesterfield, Derbyshire, UK

## Objectives

This paper will explain how morphological diversity, not corresponding to a hypothetical ‘ideal’ anatomical foot alignment, should not be regarded as representations of deformity. An alternative populationist approach to describing the foot is proposed.

## Content

The use of sub-talar joint positioning to describe intrinsic foot ‘deformity’ is principally used by podiatrists attempting to rationalise lower limb symptoms and the use of foot orthoses. In order to have evolved the physical structures that we exhibit, natural variation of form must exist, along with the conservation of common functional attributes. Results from molecular genetics research can be used to explain this process. It will be shown that regulatory epigenetic factors including homogeneic genes act together with adaptive plasticity of the weight bearing foot to produce a variety of observable physical traits. Anthropological and genetic evidence can be thus be used to redefine human foot morphology within ranges of natural continuous variation. Morphometric analysis of these traits delivers a more inclusive definition of normal alignment and can assist practitioners to focus on the universal functional characteristics of the human foot.

## Relevance

Podiatrists have been furnished with an ‘Essentialist’ approach to describing foot morphology. Deviation from a single positional ‘normality’ has wrongly been used to quantify orthotic interventions. A populationist approach offers a scientifically valid tolerance of anatomical variance and focuses specificity of orthotic interventions on appropriate phenotypes.

## Participant outcomes

A required shift in the appreciation of phenotypic variation in the treatment and research of biomechanical foot dysfunction will be conveyed. This change in philosophical approach offers a parsimonious explanation of why dissimilar patients fare better with different orthotic models.

